# Cytochrome P450-Mediated Phytoremediation using Transgenic Plants: A Need for Engineered Cytochrome P450 Enzymes

**DOI:** 10.4172/2157-7463.1000127

**Published:** 2012

**Authors:** Santosh Kumar, Mengyao Jin, James L Weemhoff

**Affiliations:** School of Pharmacy, University of Missouri, USA

**Keywords:** Phytoremediation, Transgenic plants, Cytochrome P450, P450 engineering

## Abstract

There is an increasing demand for versatile and ubiquitous Cytochrome P450 (CYP) biocatalysts for biotechnology, medicine, and bioremediation. In the last decade there has been an increase in realization of the power of CYP biocatalysts for detoxification of soil and water contaminants using transgenic plants. However, the major limitations of mammalian CYP enzymes are that they require CYP reductase (CPR) for their activity, and they show relatively low activity, stability, and expression. On the other hand, bacterial CYP enzymes show limited substrate diversity and usually do not metabolize herbicides and industrial contaminants. Therefore, there has been a considerable interest for biotechnological industries and the scientific community to design CYP enzymes to improve their catalytic efficiency, stability, expression, substrate diversity, and the suitability of P450-CPR fusion enzymes. Engineered CYP enzymes have potential for transgenic plants-mediated phytoremediation of herbicides and environmental contaminants. In this review we discuss: 1) the role of CYP enzymes in phytoremediation using transgenic plants, 2) problems associated with wild-type CYP enzymes in phytoremediation, and 3) examples of engineered CYP enzymes and their potential role in transgenic plant-mediated phytoremediation.

## Introduction

Cytochromes P450 (CYP) enzymes are a super family of ubiquitous heme proteins that are involved in Phase I metabolism and clearance of numerous xenobiotics, such as therapeutic drugs, substances of abuse, herbicides, and industrial contaminants [1]. Over 80% of marketed drugs are converted into relatively hydrophilic compounds by CYP enzymes in the liver leading to their safe clearance from the body. Although CYP enzymes are mainly involved in detoxification of xenobiotics, several CYP enzymes also activate xenobiotics, such as tobacco constituents and industrial pollutants, into toxic or carcinogenic compounds [2]. For example, CYP2A6 and CYP2A13 activate tobacco constituents, namely nitrogen-derived nitrosamines, into procarcinogens, which cause lung, esophageal, and pancreatic cancers [3]. In addition to xenobiotic metabolism/activation, CYP enzymes metabolize endogenous compounds, such as fatty acids and vitamins, and play an important role in homeostasis of these compounds [4,5].

The catalytic versatility and substrate diversity of CYP enzymes have led to considerable interest in utilizing them as biocatalysts for biotechnology (synthesis of drugs and other chemicals), medicine (biosensors and targeted gene therapy), and bioremediation (detoxification of industrial and environmental pollutants) [6–8]. In the last decade there has been an increasing realization of the power of CYP biocatalysts for creating herbicide-resistant plants and for the detoxification of soil and water contaminants using phytoremediation [7,9–11]. The use of transgenic plants for phytoremediation is critical because plants do not have the ability to completely catabolize toxic compounds that are common in the food chain, such as herbicides, pharmaceuticals, petrochemicals, polycyclic aromatic hydrocarbons (PAHs), and polychlorinated benzene (PCBs) [12,13]. Since many mammalian CYP enzymes have the capability to metabolize these compounds into relatively safe products, they can be used to create transgenic plants for such purposes. However, the major limitation of mammalian CYP enzymes is that they require the redox partner cytochrome P450 reductase (CPR), which transfers electrons from the NADPH to the heme of CYP, to oxidize the substrate [1]. In addition, mammalian CYP enzymes have relatively low turn-over (1–25), enzyme stability, and expression in heterologous systems, including plants [1,7]. In contrast, many bacterial CYP enzymes do not require external redox partner for the transfer of electrons from the NADPH to CYP, rather they contain reductase domain within the CYP enzymes (self-sufficient CYP). In addition, these enzymes have much higher turn-over (>100) and enzyme stability than the mammalian CYPs [14,15]. However, unlike mammalian CYP enzymes, bacterial CYP enzymes do not show substrate diversity and metabolize limited number of compounds [1,14]. Therefore, there is a critical need to design mammalian CYPs to improve their catalytic efficiency, stability, expression, and the suitability of P450-CPR fusion enzymes, as well as to design bacterial CYPs for enhanced stability, expression, and substrate diversity [6–8].

## Cytochrome P450-mediated Phytoremediation

### Environmental contaminants

Herbicides provide labor-saving ways of improving the yield and quality of crops, which increase the profitability and marketability of agricultural products (Table 1). However, regular use of herbicides poses a serious concern for food safety and leads to retention of these herbicides in the soil causing contamination of underground water, which is used as drinking water in many parts of the world [16,17]. Similarly, many industrial pollutants, such as PAHs, PCBs, petroleum compounds, as well as sewage disposal containing potentially toxic compounds, are the leading causes of soil, water, and air contaminations [18,19]. Further, explosive compounds, such as trinitrotoluene, hexahydro-1,3,5-trinitro-1,3,5-triazine (RDX), and chlorinated solvents (tricholorethane), are regularly disposed in the land causing environmental contamination [20]. Many countries like China and India encounter challenges in controlling the release of contaminants from industries and sewage into water and land [21, 22]. However, there have been very few attempts to detoxify these contaminants. Utilization of decontaminated soil for agriculture has significant impact in countries like Europe and Asia where there is scarcity of land. In the United States of America, the Environmental Protection Agency (EPA) has identified improvements of air and water quality as one of the top priorities (www.epa.gov).

### Plant CYPs

In addition to chemical methods, a few approaches have been taken to remove these herbicides and industrial contaminants by plant CYP enzymes [12,13]. Plant CYPs catalyze herbicide metabolism and contribute to detoxification or activation of other agrochemicals in crop plants [12,13]. However, molecular information regarding the metabolism of herbicides by plant CYPs is limited, and most herbicide-detoxifying CYPs are expressed at low levels in plants. Therefore, characterizations of plant CYP enzymes may help increase their expression levels followed by creation of herbicide-resistant plants. The first two plant CYPs that were identified are CYP76B1 from *Helianthus tuberosus* and CYP71A10 from soybean. These CYP enzymes actively metabolize herbicides, namely phenylureas [23,24]. Further, CYP81B2 and CYP71A11 were isolated from tobacco, which were shown to metabolize chlortoluron [25]. Although these CYP enzymes play some role in herbicide metabolism, these plants do not contain a complete and efficient catabolic pathway to detoxify herbicides and other toxic compounds [12,13,23–25]. Therefore, bacterial and mammalian CYP enzymes have been introduced in various plants for effective phytoremediation of these chemical [9–11].

### Bacterial CYPs

Transgenic plants expressing bacterial CYP genes have been used for enhanced degradation and remediation of herbicides and industrial contaminants [9–11]. In a revealing example, *Arabidopsis thaliana* expressing *Rhodococcus rhodochorus* xplA (fused to a flavodoxin redox partner) degraded RDX grown in RDX-contaminated soil and was found to be resistant to RDX phytotoxicity [26]. The transgenic plants also produced shoot and root biomasses that were greater than those of wild-type plants. Thus, transgenic plants expressing xplA may provide a phytoremediation strategy for sites contaminated by this class of explosives [27]. In another example, CYP105A1 from *Streptomyces griseolus* was cloned in tobacco plants for the degradation of sulfonylurea pro-herbicide [28]. In this case, the expressed CYP105A1 appeared to interact with the host redox partner present in the chloroplast for the metabolism of sulfonylureas.

### Mammalian CYPs

A comprehensive study by Inui and colleagues has shown that human CYP1A1 can metabolize 16 herbicides, CYP2B6 can metabolize more than 10 herbicides, three insecticides, and two industrial chemicals, and CYP2C19 can metabolize 16 herbicides [29]. Similarly, human CYP1A1 and CYP1A2 can metabolize PAHs, while CYP2B6 and canine CYP2B11 can metabolize PCBs [1,30]. Further, CYP2E1 can metabolize volatile organic compounds, such as chloroform, and carbon tetrachloride [31]. Therefore, creating transgenic plants using human CYP enzymes has the potential for developing herbicide-resistant plants, as well as for reducing the environmental impacts of soils, water, and air pollutions. In the past decade CYP1A1, CYP2B6, CYP2B22, CYP2C9, CYP2C19, CYP2C49, CYP2E1, CYP71A1, CYP76B1, and CYP105A1 have been introduced in various plants, such as rice and tobacco, for phytoremediation of numerous herbicides, petrochemicals, and industrial contaminants [31–34]. For example, transgenic rice (*Oryza sativa*) plants that harbor CYP1A1, CYP2B6, and CYP2C19 genes were more tolerant to several herbicides than non-transgenic plants [32–33]. The transgenic rice plants, as a result of the CYP activity, showed enhanced herbicide metabolism and was able to remove atrazine, norflurazon, metolachlor, and sulfonylurea from the soil.

Similar to the Phase 2 drug metabolism in the liver [1], herbicides and industrial pollutants also require Phase II enzyme, namely glutathione S-transferase (GST), for their complete degradation. Therefore, transgenic tobacco plants expressing maize GST1 were developed for enhanced phytoremediation of chloroacetanilide [35]. Similarly, GST-dependent detoxification of many organic xenobiotics, such as acetylsalicylic acid, paracetamol, 1-chloro-2, 4-dinitrobenzene, and propachlor, were obtained using transgenic *Phragmites australis* [36]. Recently, human CYP2E1 and fungal GST were simultaneously cloned in *Nicotiana tabacum*, which showed enhanced degradation of anthracene and chloropyriphos [37]. It is expected that the transgenic expression of both Phase I (CYP) and Phase II (GST) enzymes in plants will provide enhanced and complete detoxification of organic xenobiotics.

Thus, CYP-based transgenic plants have shown potential in detoxifying soil contaminants and improving the health of the plants. The successful implementation of this technology on a large scale has the potential to improve agricultural production, increase land area that is currently unused due to pollution, and improve the quality of underground water. However, the wild-type mammalian and bacterial CYP enzymes have inherent limitations, which pose obstacles in effective phytoremediation.

## Problems Associated with Wild-type Cytochrome P450 Enzymes in Phytoremediation

Although bacterial CYP enzymes are self-sufficient and show high activity, they are not suitable for detoxification of many herbicides or industrial contaminants because they have limited capability to metabolize these xenobiotics [14,15]. Thus, it will be a daunting task to engineer bacterial CYP enzymes, which have a relatively rigid and small active site(s), and metabolize relatively small compounds, for the metabolism of large compounds including herbicides. On the other hand, generating transgenic plants using bacterial CYPBM3, which are capable of metabolizing a variety of gasoline, have potential in phytoremediation of petroleum pollutants [7,14]. CYPBM3 can be engineered to metabolize novel petroleum substrates that are of similar sizes and shapes, which could further be used for phytoremediation of a variety of petroleum compounds [14,38].

The major challenge that limits the successful use of mammalian CYPs in phytoremediation is that they have lower activity and stability than bacterial CYP enzymes. In addition, mammalian CYPs require CPR for the transfer of electrons from NADPH to the heme of CYP for their activity, which is difficult to control in transgenic plants. However, since mammalian CYPs have the ability to metabolize a wide range of substrates, including herbicides and environmental contaminants, the engineering of mammalian CYP enzymes with increased activity can produce CYP biocatalysts that are amenable to phytoremediation [1,7]. Mammalian CYPs can also be engineered using artificial fusion proteins that would couple between CYP and CPR for efficient transfer of electrons.

**Figure 1:**
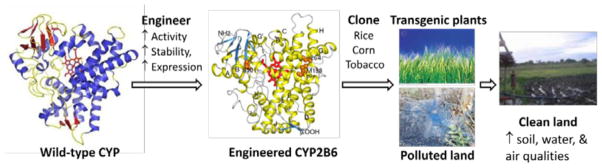
Engineering of CYP enzymes for improved activity, stability, and expression using rational and/or directed evolution approaches. Cloning of engineered CYP enzymes (e.g. CYP2B6) in plants followed by growth in polluted lands has the capability to detoxify the lands, leading to increased production of crops and increased air and water qualities.

CYP enzymes can be engineered by using rational (knowledge-based) and/or irrational approaches [7,39]. Rational approach can be used upon knowledge obtained from primary sequence and structure-function analysis of CYP enzymes. However, irrational approach using directed evolution can be employed to engineer CYP enzymes when their structure-function relationships is lacking. Directed evolution approach for the engineering of CYP enzymes is necessary because many non-active site residues, which are not yet characterized, have been shown to control enzyme activity, stability, and expression in heterologous systems [7,39]. Several CYP enzymes have been engineered using both approaches, which could drive the use of these or newly engineered CYP enzymes for phytoremediation using transgenic plants [6,7,39]. Figure 1 summarizes the engineering of CYP enzymes for enhanced activity, stability, and expression using the rational or directed evolution approach followed by cloning of the engineered CYP enzymes in plants that can be used to detoxify polluted lands.

## Engineering of Cytochrome P450 Enzymes and their Potential Application in Phytoremediation

### Bacterial CYPs

In the past decade, significant progress has been made in designing bacterial CYP enzymes using rational and directed evolution approaches for enhanced expression, catalytic activity, thermo stability, cofactor requirements, and solvent tolerance [6,7,40–43] (Table 2). In a revealing finding, a ω-fatty acid metabolizing CYPBM3, upon engineering using directed evolution, showed enhanced activity towards hydroxylation of many non-natural alkanes, such asoctane, hexane, propane, butane, and ethane [41]. The engineered enzymes exhibited higher activity than any reported biocatalyst for the selective oxidation of hydrocarbons of small to medium chain length (C3–C8). Furthermore, CYPBM3 was engineered for increased activity with other non-alkyl substrates, such as benzene, propyl benzene, and naphthalene [43]. Many of these engineered CYPBM3 enzymes have shown applications in bioremediation, such as detoxification of terpenes and gaseous alkanes [14,40].

CYPCAM (CYP 101) has been engineered using rational approach for efficient oxidation of alkanes, halogenated hexanes, PCBs, PAHs, and unnatural substrates [44–47]. The engineered CYPCAM enzymes showed enhanced activity towards linear alkanes such as butane, pentane, hexane, and heptanes [44]. They also showed an enhanced activity with PAHs and PCBs [45–46]. Furthermore, CYPCAM was engineered for the increased oxidation of non-natural substrates; diphenylmethane, styrene, and ethylbenzene [47]. Recently, CYPCAM was engineered by directed evolution via a step-by-step adaptation to smaller alkanes, from hexane to ethane [48]. The engineered CYPBM3 and CYPCAM enzymes can potentially be used to create transgenic plants for possible phytoremediation of these toxic compounds. These engineered enzymes can be tested to examine whether they also show enhanced activity towards herbicides and other environmental contaminants. Furthermore, they can specifically be designed to metabolize herbicides, as well as other important petroleum aliphatic and aromatic hydrocarbons.

### Mammalian CYPs

Although it is relatively difficult to engineer mammalian CYP enzymes compared to bacterial enzymes, the engineering of mammalian CYP enzymes is of high priority. *Guengerich* and colleagues have successfully engineered CYP1A2 and CYP2A6 for enhanced activity with several known, as well as novel substrates, e.g. CYP1A2 for alkoxy resourin compounds and CYP2A6 for indole compounds [49,50]. They used rational and directed evolution approaches to engineer these enzymes. They also generated transgenic tobacco plants harboring engineered CYP2A6, which produce the precursor of indole dyes [51]. Similarly, Kumar and Halpert groups have engineered several mammalian CYP2B enzymes for enhanced activity, expression, stability, and substrate specificity by using rational and directed evolution approaches [52–55]. Rat CYP2B1 and canine CYP2B11 were engineered for enhanced expression and stability, as well as increased activity with several compounds, including 7-ethoxytrifluorocoumarin and the anti-cancer prodrugs cyclophosphamide and ifosfamide [52,53]. Human CYP2B6 was engineered for enhanced expression and stability, while human CYP3A4 was engineered for enhanced activity with several substrates, including 7-benzyloxy-trifluoro-coumarin and testosterone [54,55]. Furthermore, Gillam and colleagues applied the directed evolution approach to design CYP2C enzymes, which showed distinct and novel activity with many substrates, such as 6′-deoxyluciferin and diclofenac [56]. Although these mammalian CYP enzymes were not engineered for enhanced activity with herbicides, industrial contaminants, or petroleum compounds, this provides a proof-of-concept that mammalian CYP enzymes can also be engineered for bioremediation.

The application of engineered mammalian CYP enzymes for phytoremediation has not yet been realized, apparently, because upon engineering there were relatively small increases in activity, and most of the increased activities were towards non-pollutants and non-herbicides. [6,7]. Although expression of engineered enzymes in bacterial system was significantly increased, there was no attempt to express them in plants. We suggest a potential use of engineered mammalian CYP enzymes in bioremediation that show significantly increased activity, substrate diversity, and expression. Thus, mammalian CYPs (CYP1A1, CYP1A2, CYP2B6, and CYP2B11) that are known to metabolize several herbicides and industrial pollutants [1,7,29] and that have already been engineered for enhanced activity [49–56], need to be tested for their enhanced activity with herbicides and industrial pollutants. The selected CYPs that show enhanced activity with these compounds can be used to generate transgenic plants for phytoremediation. Subsequently, these CYP enzymes can further be designed for enhanced metabolism of herbicides and industrial pollutants and for increased expression in plant systems.

Finally, we suggest the application of artificial fusion proteins by improving the coupling between mammalian CYP and CPR enzymes and exploiting alternative electron transport partners [57]. In most cases, the catalytic rates with fusion enzymes are not optimal, and therefore, further improvement in creating the fusion enzymes is needed. Since fusion enzymes still require the expensive cofactor NADPH, alternative means, which utilize light-driven NADPH synthesis in chloroplast and engineering CPR for increased specificity towards NADH, have been proposed [57]. A co-expression system also appears to be the most effective in generating transgenic plants for phytoremediation. Alternatively, for phytoremediation, CYPs need to be engineered to interact with the host cell reductase(s) and for their subcellular and tissue distributions.

## Conclusion

Bioremediation of soil contaminants arising from herbicides and industrial pollutants has been identified as critically important in order to increase the production of crops, usage of unused contaminated land, and improvement in the quality of underground water. Generation of CYP-based transgenic plants have the potential to serve this purpose. However, certain limitations of CYP enzymes need to be overcome prior to their use in phytoremediation. Therefore, there is a critical need to design CYP enzymes to overcome their limitations in order to make them amenable for phytoremediation of herbicides and industrial pollutants. Successful approaches towards this will provide us an opportunity to clean up our environment, leading to improved crop production and quality, and improved water and air qualities.

## Figures and Tables

**Table 1:** Summary of potential use of the wild-type CYP enzymes in phytoremediation of herbicides and pollutants.

Source	CYP enzymes (*source type*)	Substrates
Plant	CYP76B1 (*H. tuberosus*)	Phenylureas
	CYP71A10 (soybean)	Phenylureas
	CYP81B2, CYP71A11 (tobacco)	Chlortoluron
Bacterial CYPs in transgenic plants	xp1A (*R. rhodochorus*)	Hexahydro-1,3,5-trinitro-1,3,5- trizine (RDX)
	CYP105A1 (*S. griseolus*)	Sulfonylureas
Mammalian CYPs in transgenic plants	CYP1A1 (human)	Herbicides
	CYP2B6 (human)	Herbicides and insecticides
	CYP2C19 (human)	Herbicides
	CYP2E1 (human)	Volatile organic compounds

**Table 2:** Summary of the engineered CYP enzymes for the metabolism of various substrates.

Source	CYP enzymes	Substrates
Bacterial CYPs	CYPBM3	Alkanes (octane to ethane), gaseous alkanes, benzene, propyl benzene, naphthalene
	CYPCAM	Alkanes (heptane to ethane), halogenated hexanes, PAHs, PCBs, styrene, diphenylmethane, ethylbenzene
Mammalian CYPs	CYP1A2	Alkoxyresorufin compounds
	CYP2A6	Indole compounds
	CYP2B1	7-ethoxytrifluorocoumarin, cyclophosphamide, ifosfamide
	CYP2B11	7-ethoxytrifluorocoumarin, cyclophosphamide, ifosfamide
	CYP2Cs	6′-deoxyluciferin, diclofenac
	CYP3A4	7-benzyloxytrifluorocoumarin, testosterone
